# Knowledge, attitudes and practices pertaining to urogenital schistosomiasis in Lambaréné and surrounding areas, Gabon

**DOI:** 10.1186/s13071-021-04905-0

**Published:** 2021-09-22

**Authors:** Jean Claude Dejon-Agobé, Jeannot Fréjus Zinsou, Yabo Josiane Honkpehedji, Jean Ronald Edoa, Bayodé Roméo Adegbité, Romuald Beh-Mba, Peter Gottfried Kremsner, Ayola Akim Adegnika, Martin Peter Grobusch

**Affiliations:** 1 Centre de Recherches Médicales de Lambaréné (CERMEL), Lambaréné, Gabon; 2 Center of Tropical Medicine and Travel Medicine, Department of Infectious Diseases, Amsterdam Infection and Immunity, Amsterdam Public Health, Amsterdam University Medical Center, location AMC, University of Amsterdam, Amsterdam, The Netherlands; 3 Department of Parasitology, Leiden University Medical Center, Leiden, The Netherlands; 4 Fondation pour la Recherche Scientifique, 72 BP45 Cotonou, Bénin; 5 Institut Für Tropenmedizin, Eberhard Karls Universität Tübingen, Tübingen, Germany; 6 German Center for Infection Research (DZIF), Tübingen, Germany; 7 Masanga Medical Research Unit, Masanga, Sierra Leone; 8 Institute of Infectious Diseases and Molecular Medicine, University of Cape Town, Cape Town, South Africa

**Keywords:** Knowledge, Attitude, Practice, Schistosomiasis, Risk-enhancing practices, Lambaréné, Gabon

## Abstract

**Background:**

Control of schistosomiasis remains a priority in endemic areas. Local epidemiological data are necessary for a tailored control programme, including data on population behaviour in relation to the disease. The objective of this study was to assess schistosomiasis-related knowledge, attitudes and practices in the general population of Lambaréné, a small city in Gabon, in order to optimise the design and implementation of a local control programme that is tailored to need.

**Methods:**

The study was cross-sectional in nature. Eligible adults and children living in the study area who volunteered (with informed consent) to participate in the study were interviewed using standardised questionnaires, one of which was a simplified version of the primary questionnaire for participants aged 6–13 years. Data on the participants’ knowledge, attitudes and practices that enhance the risk for contracting schistosomiasis were collected.

**Results:**

A total of 602 participants were included. The mean (± standard deviation) age was 21.2 (± 15.0) years, the female:male gender ratio was 1.6 and 289 (48%) participants completed the simplified version the questionnaire. Of the 602 participants, 554 (92%) reported past or current contact with freshwater, 218 (36%) reported a history of a diagnosis of schistosomiasis and 193 (32%) reported past intake of praziquantel medication. The overall levels of knowledge and adequate attitudes toward schistosomiasis among young adults and adults were 68 and 73%, respectively. The proportion of participants pursuing risk-enhancing practices (REP) was 60% among the whole study population. Location was significantly associated with differences in knowledge and REP levels. A history of confirmed schistosomiasis and larger family size were significantly associated with an increase in good knowledge and REP levels. However, the indication of freshwater-associated activities was only associated with a significant increase in the REP level.

**Conclusions:**

The results of this survey reveal a high level of population exposure to schistosomiasis, which is in line with known prevalence of schistosomiasis in Lambaréné and its surroundings. The local population has a reasonable level of knowledge of and adequate attitudes toward schistosomiasis but the level of REP is high, particularly in areas where piped water is absent. In terms of interventions, improving hygiene should have the highest priority, but in a context where provision of safe water is difficult to achieve, the effectiveness of praziquantel treatment and the education of at-risk populations on the need for protective behaviours should be a prominent feature of any local control programme.

**Graphical abstract:**

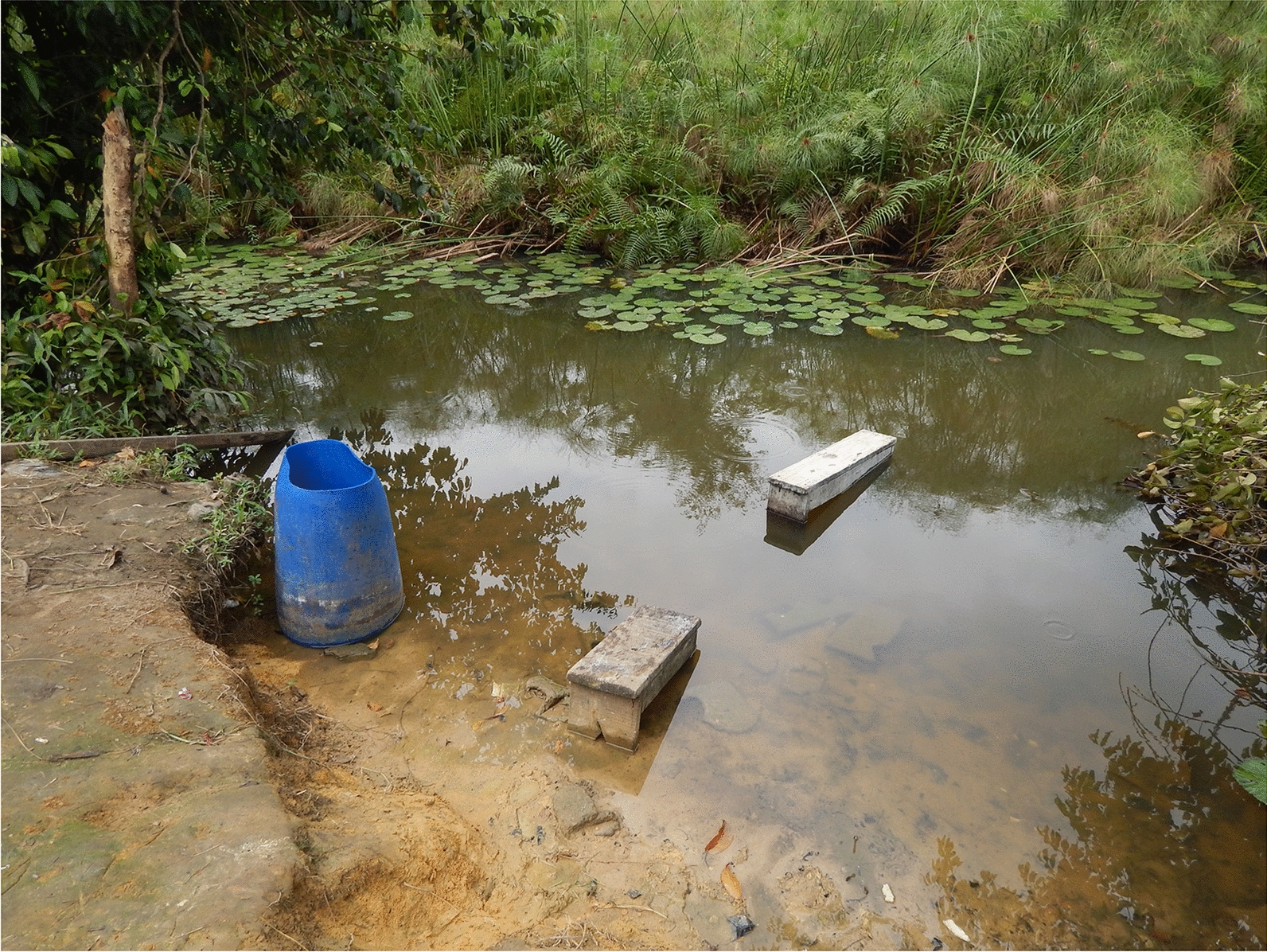

**Supplementary Information:**

The online version contains supplementary material available at 10.1186/s13071-021-04905-0.

## Background

Schistosomiasis ranks only second to malaria amongst the most devastating human parasitic diseases [1] and remains a public health threat in several parts of the world. This is particularly the case in sub-Saharan Africa where schistosomiasis is the most deadly neglected tropical disease (NTD) after snakebite envenomation, killing an estimated 2,80,000 people each year [1]. Schistosomiasis is especially prevalent in poor communities with limited access to safe water and adequate sanitation [2] and mostly affects communities exposed to these conditions, particularly agricultural and fishing populations, as well as women doing domestic chores involving infested water, such as washing clothes and dishes. Inadequate hygiene and contact with infested water render children in particular vulnerable to infection [2].

Schistosomiasis is a water-borne disease. Human–water contact is therefore an important factor to consider when disease control programmes are being designed. In addition to mass drug administration (MDA) and snail control, the World Health Organization (WHO) strategy for schistosomiasis control is based on the access to safe water (W), improved sanitation (S) and hygiene education (H) of at-risk population groups [2], a strategy known as the WASH programme [3]. However, in a context where achieving access to safe water sources may be difficult, hygiene education becomes the cornerstone of WASH. It has been demonstrated that the implementation of education of at-risk populations can considerably improve the control of schistosomiasis, even in highly endemic areas [4, 5]. In order to propose the appropriate change in behaviour or better adaptative behaviour, and to implement an adequate approach for prevention, it thus becomes necessary to assess the knowledge of the local population in each area where the disease is endemic, and also to identify the attitudes and practices (KAP) of these local populations toward the disease. To this end, the KAP survey appears to be a relevant tool to explore the local situation, as a pre-requisite for targeting group-tailored intervention strategies. A number of previous KAP studies on schistosomiasis that were conducted in rural and semi-urban areas, where sanitation is usually rudimentary and safe water supply is limited, demonstrated that even when the levels of knowledge and attitude in relation to schistosomiasis are moderate or good [6, 7], disease prevention often remains difficult to practise - mainly in terms of personal and sanitation hygiene, in both children [6, 7] and adults [7] with limited access to adequate toilet facilities and safe water, but also possibly in combination with a lack of knowledge of how schistosomiasis is transmitted or could be prevented [8].

Lambaréné is the capital town of Moyen-Ogooué, one of Gabon’s nine provinces. At the time of our study, no previous KAP study on schistosomiasis had been conducted in Lambaréné and its surrounding rural areas; thus, there was a lack of important and relevant information. We report here the results of a KAP study that was performed to gain insight into the knowledge, attitudes and practices of local people regarding schistosomiasis, with the goal to facilitate control program optimisation, as the region appears to be one of the areas of the country worst affected by schistosomiasis.

## Methods

### Study design

The study was cross-sectional in design, with eligible volunteers invited to participate. Participants who agreed to partake in the study were asked to take part in individual interviews. The data obtained during these interviews were captured on a standardised questionnaire. Because the targeted population was homogenous, no focus group interviews were conducted.

### Study site

Lambaréné is a semi-urban area located by road 240 km south of Libreville, the capital of Gabon, and 110 km south of the equator. It is the capital city of the region and hosts the administrative institutions. The city is located on an island in the Ogooué River and its tributaries, and there are many ponds, lakes and streams in the city. The municipal water supply network does not cover all areas of the town. The area is endemic mainly for *S. haematobium* [9–11]. The municipal water supply network does not cover all areas of town. Lambaréné is surrounded by rural areas all endemic for S. haematobium [9, 10] mainly, namely Zilé-PK and Mitonè-PK areas. The Zilé-PK area includes a set of villages located south of Lambaréné along the first national road (RN1) from km 12 to 33 km, while the Mitonè-PK area includes a set of villages extended over 30 km along RN1 to the north of Lambaréné. Both areas are characterised by basic, rudimentary sanitary facilities and the lack of an adequate water supply network. However, each major village in Zilé-PK area has a public pump, although the streams and tributaries of the Ogooué River still constitute the preferable source of water for household activities and bathing. An industrial palm oil plantation is also located in Zilé-PK area, in the proximity of the RN1 (about 500 m) at the PK 15 level; this plantation provides housing to their workers, including electricity and a number of points of piped water in addition to the freshwater used by the inhabitants. The whole locality has four primary schools and two dispensaries. Additional study locations were Bindo and Makouké, two remote rural settlements about 65 km by road north of Lambaréné. Both areas are located on the banks of the Ogooué River and are surrounded by an industrial palm oil plantation that provides accomodations for its workers, including housing, basic facilities for hygiene, water and electricity. In Bindo, water is supplied directly from the Ogooué River to two public taps through a pipeline; in Makouké, water is treated before reaching the public tap or the houses directly. Each locality has one primary school and one dispensary with one nurse from the Bindo site and a medical team led by one medical doctor from the Makouké site. Makouké also has one secondary school, which is attended by children from both Makouké and Bindo. Both localities are linked by a ferry across the Ogooué River.

Only a few MDA campaigns on the control of schistosomiasis have been conducted by the government in these areas. According to the local health authorities, the last praziquantel (PZQ) treatment campaign took place among school children in 2016. No schistosomiasis awareness campaigns were run in the area.

### Study population

The Lambaréné population is about 45,000 inhabitants [12], and Bindo-Makouké and the surrounding areas of Lambaréné (Zilé-PK and Mitonè-PK areas) have about 1000 and 2500 inhabitants, respectively [9]. Agriculture and fishing are the main activities of the young population from the surrounding areas, while it is less practiced by residents of Lambaréné and Bindo-Makouké; rather, the populations of Bindo and Makouké are essentially workers on the local palm oil plantation. From the three areas, volunteers, including school-age children and adults, were invited to participate in the study. However, individuals unable to respond to the questionnaire were excluded.

### Questionnaire

The main standardised questionnaire (Additional file 1: Text S1) used in this study was developed using Médecins du Monde [13] and Handicap International [14] guidelines and designed to collect data on the knowledge, attitudes (including prevention strategies) and risk-enhancing practices (REP) regarding schistosomiasis through closed questions among participants aged ≥ 14 years (young adults and adults). In order to allow the interviewee to give his/her position on some aspect of the topic, open questions were also asked. For volunteers aged < 14 years, we used a simplified version of the questionnaire that corresponded with the main questionnaire. This simplified version had been developed for children aged 6 to 13 years from the main study questionnaire by removing inappropriate questions for age, and simplifying the language of the remaining questions. In addition, some illustrating images were added to brighten up the questionnaire and facilitate comprehension of the questions (Additional file 2: Text S2). For validation, the simplified version was pretested in the corresponding study population before being used in the survey. The simplified questionnaire thus mainly assessed the REP of children with regard to schistosomiasis. In addition, elementary knowledge and attitude of these children were assessed. Both questionnaires were used in accordance with the participant’s age and were adjusted accordingly by removing questions inappropriate for age, or by simplifying the language.

### Calculation of sample size and sampling

Awareness of schistosomiasis was considered to be the primary indicator ofe population knowledge on schistosomiasis, and we assumed that 75% of the local population was aware of schistosomiasis. With 95% confidence level and a standard error of 5%, we needed at least 288 volunteers to be able to address our main objective, using the formula for cross-sectional study sample size calculation as described elswhere [15]. Given that two different questionnaires were used, we considered a calculated sample size for each questionnaire, giving a total of 576 volunteers in total to include in the study. These volunteers were selected from the different study areas by applying a three-stage sampling procedure. The first stage included random selection of a neighbourhood or village from each area. The second stage included the selection of households from these neighbourhoods or villages through systematic sampling, with the third inhabited household systematically selected from every three households. At the third stage, a maximun of two respondents was randomly selected from each selected household.

### Data collection

The survey was conducted from June to July 2019. Data on knowledge, attitude and practices were gathered through the standardised questionnaire. Socio-demographic data were collected using the same questionnaire, as were clinical data on schistosomiasis and data on history of taking PZQ. The questionnaire was given in the national language, French, to each respondent included in the study during an approximately 15-min face-to-face interview. The simplified version of the questionnaire was used with participants aged 6–13 years. In order to minimise inter-interviewer differences, a (re)training session was organised every three days of data collection. Data were collected using the paper version of the questionnaire and then digitalised with REDCap software [16] hosted at CERMEL (Centre de Recherches Médicales de Lambaréné). The clean database was extracted and imported in R software version 3.4.4 (R core team, Vienna, Austria) for analysis.

### Statistical considerations

We were interested in assessing the knowledge, attitudes and practices of the study population regarding schistosomiasis, and these variables were considered to be the variables of interest. Other variables included in the analysis were considered as explanatory variables. In the descriptive analysis, we reported results as a proportion of answers, while for the analysis of association, a score was calculated for each variable of interest, as the total score for each appropriate answer related to that variable. For questions on knowledge and attitudes, a score of one point was given for each correct answer considered as appropriate while for questions on REP, a score of one point was given for each answer indicating a risk of contamination with schistosomiasis. For closed questions with multiple ordinal responses, an additional score of one point was added at each modality level from zero corresponding to the wrong knowledge or attitude, or for absence of risk practices, amounting to potential maximum scores of 13, 4 and 9 points for knowledge, attitudes and REP, respectively (Additional file 3: Table S1). The overall level of appropriate knowledge, attitudes and REP were estimated as the percentages of total scores. The level of this percentage was classified as ‘bad’ if < 50%; ‘acceptable’ if < 60%; ‘fairly good’ if < 70%; ‘good’ if < 80%; ‘very good’ if < 90%; and ‘excellent’ if ≥ 90%. The majority of the questions on knowledge were deemed inappropriate for children aged 6–13 years. In the simplified version of the questionnaire, we thus collected only basic information on the knowledge of children on schistosomiasis; consequently, we could not estimate the level of children’s knowledge in the same manner as we did for adults, and we therefore did not include children in the multivariate analysis shown in Table 5. The situation was different for the evaluation of REP in children. Indeed, as for young adults and adults, we were able to estimate the level of REP in children and, therefore, the regression analysis for REP includes the complete study population.

The scores were used as quantitative variables in the comparative analysis. Quantitative variables were described as the mean and standard deviation (SD) while categorical variables were described as proportions with a 95% confidence interval (CI). Linear regression was used to assess the association between the variables of interest, and demographic, socio-economic and clinical variables related to schistosomiasis were considered as explanatory variables. For multivariable regression, the final model included all variables known to be associated with schistosomiasis and considered in ther univariable analysis. The significance of statistical tests was set at < 0.05.

## Results

### Study population

A total of 304 households were visited across the study area, with a total of 1799 inhabitants aged ≥ 6 years considered to be potential eligible candidates to participate in the study. Among these, 644 volunteers were randomly selected to participate in the study, and 602 (93%) subsequently consented to be included in the survey. The 42 inhabitants who refused to participate mentioned either a lack of time or just not wanting to be involved in the survey. As all data were collected after informed consent, we could not further test differences between those who consented and those who did not. The mean  (± SD) age of the overall study population was 21.2 ± 15.0 years, with a 1.6 female:male gender ratio. As shown in Table 1, the majority of the participants came from downtown Lambaréné (*n* = 329, 55%), while almost all of them had obtained either primary (53%) or secondary (43%) educational level. As the main occupation, 84 (27%) participants among the 313 who responded to the main questionnaire were students, 69 (22%) were farmers and/or fishermen, and 75 (24%) reported to have no main occupation. From the whole study population, 313 (52%) responded to the comprehensive study questionnaire, while 289 (48%) participants responded to the simplified version of the questionnaire. Of those who responded to the simplified version of the questionnaire, 280 (97%) were school children.

**Table 1:** Sociodemographic characteristics of the 602 study participants (entire study population)

Sociodemographic characteristics	Study population		
	*n*	%	95% CI (%)
Age (in years)			
6–9	117	19.4	16.3–22.8
10–13	128	21.3	18.2–24.9
14–17	91	15.1	12.5–18.4
18–25	102	17.0	14.2–20.4
> 25	164	27.3	23.9–31.2
Gender			
Female	368	61.1	57.1–65.0
Male	234	38.9	35.0–42.9
Location			
Lambaréné	329	54.7	50.6–58.7
Mitonè-PK villages	52	8.6	6.5–11.2
Zilé-PK villages	154	25.6	22.1–29.3
Bindo-Makouké villages	67	11.1	87.3–13.9
Educational level			
None	13	2.2	1.1–3.7
Primary	318	52.8	48.7–56.9
Secondary	262	43.5	39.5–47.6
University	6	1.0	0.4–2.2
Other	3	0.5	0.1–1.4
Main occupational status^a^			
None-working	75	24.0	19.3–29.1
Farmer or fisher	69	22.0	17.6–27.0
Trader	21	6.7	4.2–10.1
Student	84	26.8	22.0–32.1
Administrative	4	1.3	0.3–3.2
Health care	6	1.9	0.7–4.1
Other	54	17.3	13.2–21.9
Family size^a^			
1–3	77	24.6	19.9–29.8
4–6	130	41.5	36.0–47.2
≥ 7	106	33.9	28.6–39.4

CI Confidence interval

^a^Applicable to young adults and adults only, corresponding to the main version of the questionnaire

### Human–water contact profile of entire study population

As shown in Table 2, 311 (52%) of the 602 participants reported having access to piped water at home for their household activities, and 284 (47%) declared using open freshwater sources. A total of 417 (69%) of the study population considered their house close to a water course; among these, 261 (62%) lived near a river, and 51 (12%), 23 (5%), 22 (5%) and 73 (17%) lived near a lake, stream, swamp or the Ogooué River, respectively. A pit latrine was used by 72% of the study population, while 11 and 18% reported using either private toilets or sharing modern and external toilets with the neighbourhood, respectively.

**Table 2:** Distribution of factors inherent in schistosomiasis among the 602 study participants

Factors associated with schistosomiasis	Study population		
	*n*	%	95% CI (%)
History of *Schistosoma* infection			
Yes	217	36.0	32.2–40.0
History of visible haematuria			
Yes	241	40.0	36.1–44.1
History of PZQ treatment			
Yes	193	32.1	28.3–35.9
Avenues of PZQ^a^			
Local research centre (CERMEL)	95	49.2	42.0–56.5
Health centre	42	21.8	16.2–28.2
From a parent	23	11.9	7.7–17.3
National campaign of MDA of PZQ	20	10.4	6.4–15.5
Drugstore	16	8.3	4.8–13.1
Other	9	4.7	2.1–8.7
Source of water at home			
Tap water	311	51.7	47.6–55.7
Stream/river	284	47.2	43.1–51.2
Well	100	16.6	13.7–19.8
Ogooué River	57	9.3	7.1–11.9
Consider their house to be near a body water			
Yes	417	69.3	65.4–72.9
Type of body water considered as being near houses^b^			
River	261	62.1	57.3–66.8
Lack	51	12.2	9.2–15.8
Stream	23	5.5	3.5–8.2
Swamp	22	5.3	3.3–7.9
Ogooué River	73	17.5	14.0–21.5
Type of toilets used at home			
Private toilets	69	11.5	9.0–14.3
Shared toilets	106	17.6	14.6–20.9
Pit latrine	437	72.6	68.8–76.1

MDA, Mass drug administration; PZQ praziquantel

^a^Assessed among the 193 participants with a history of taking PZQ

^b^Assessed among the 417 participants who consider their home as being located near a body water

### Exposure of study population to schistosomiasis

Regarding study participants’ contact with fresh water in the study area, 554 (92%) individuals reported having been in contact in one way or another with an open body of fresh water, with the majority (473/554; 85%) reporting the main contact was with some of the local small rivers, some of the local small rivers, followed by some of the local lakes (86; 15%) and the Ogooué River itself (75; 13%) (Table 3). When asked about the place closest to the freshwater body, home was reported by 538 (97%) participants, while plantation and school/place of work were reported by 74 (13%) and 21 (4%) participants, respectively. In terms of the frequency of their contact with fresh water, 281 (51%) participants reported daily contact, while 48 (9%) and 220 (40%) reported once weekly or only occasional contact, respectively. The morning was the period of the day when most of the study population was in contact with fresh water (05:00 h to 11:00 h; 51%, 281/554), followed by afternoon (15:00 h to 18:00 h; 23%, 130/554), noon (12:00 h to 14:00 h; 4%, 22/554), and night (< 1%; 19:00 h to 04:00 h; 3/554). Taking a bath (505; 91%) was the most frequently mentioned reason for freshwater contact, followed by household activities (460; 83%), fetching water (413; 74%), fishing (189; 34%), playing (148; 27%) and planting (44; 8%).

**Table 3:** Distribution of factors related to human–freshwater contact among the 554 study participants who declared having had previous contact with freshwater bodies

Factors related to human–freshwater contact	Study population		
	*n*	%	95% CI (%)
Type of water point			
River	473	85.4	82.2–88.2
Lake	86	15.5	12.6–18.8
Ogooué River	75	13.5	10.8–16.7
Stream	20	3.6	2.2–5.5
Others	6	1.1	0.4–2.3
Places from which water contact was made			
Home	538	97.1	95.3–98.3
School or place of work	21	3.8	2.4–5.7
Planting	74	13.4	10.6–16.5
Others	4	0.7	0.2–1.8
Frequency of contact			
Daily	286	51,6	47.4–55.9
Weekly	48	8.7	6.5–11.3
Some times monthly	220	39.7	35.6–43.9
Time of day contact was made with freshwater body			
Morning	281	50.7	46.5–55.0
Midday: 12:00 to 15:00 h	22	4.0	2.5–5.9
Afternoon: 15:00 h to 18:00 h	130	23.5	20.0–27.2
Night	3	0.5	0.1–1.6
No specific time	118	21.3	18.0–24.9
Reason declared for water contact			
Bath	505	91.1	88.5–93.4
Housework	460	83.0	79.6–86.1
Fetch water	413	74.5	70.7–78.1
Fishing	189	34.1	30.2–38.2
Playing	148	26.7	23.1–30.6
Planting	44	7.9	5.8–10.5
Others	0	0.0	–

### History of schistosomiasis among the study population

Table 2 includes the results on the history of schistosomiasis among the 602 participants included in the study: 241 (40%) declared having already experienced visible haematuria in the past, and 217 (36%) declared a history of schistosomiasis diagnosis. In terms of having takenf PZQ as treatment of schistosomiasis, 193 (32%) remembered having received the drug at least once in the past. The Centre de Recherches Médicales de Lambaréné (CERMEL) was found to be the main source of the drug (95; 49%), followed by local health centres (42; 22%), relatives (23; 12%), national mass PZQ administration campaign (20; 10%) and drugstores (16; 8%).

### Knowledge of schistosomiasis and associated factors

The only name known to the local population to indicate schistosomiasis is the French word ‘bilharzie’. Of the 602 participants included in the study, 475 (79%; 95% CI 75–82) had already heard of schistosomiasis. The knowledge elements presented in Table 4 were assessed among the 313 young adults and adults who responded to the main study questionnaire. Of these, 301 (96%; 95% CI 75–82) had already heard of schistosomiasis. ‘Bacteria’ (33%; 95% CI 28–38) and ‘worms’ (27%; 95% CI 23–33) were the most frequently indicated causative agent of the disease, while the river small snails (46%; 95% CI 41–52) were mainly indicated as the ‘animal’ transmitting the disease, among others listed. Blood in urine was the main disease symptom indicated (91%; 95% CI 87–94), while getting in contact with river water (70%; 95% CI 64–75) was the answer mainly indicated as the way to become infected. A total of 225 (72%: 95% CI 72–81) participants knew that the diagnosis of the disease can be made by urine examination in a laboratory. When asked about the consequences of schistosomiasis, infertility (70%, 95% CI 65–75) was the main answer selected, followed by smelly vaginal discharge in women (56%, 95% CI 50–61) and girls (53%; 95% CI 48–59), then anaemia (55%, 95% CI 49–60), cancer of the bladder (50%, 95% CI 44–55) and death (48%, 95% CI 42–53).

**Table 4:** Distribution of knowledge of schistosomiasis among the 313 young adults and adults responding the main questionnaire

Knowledge of schistosomiasis	Study population		
	*n*	%	95% CI (%)
Ever heard of schistosomiasis			
Yes	301	96.2	93.4–98.0
Indicated as causative agent of schistosomiasis			
Worm	86	27.5	22.6–32.8
Virus	46	14.7	11.0–19.1
Bacteria	103	32.9	27.7–38.4
Other	7	2.2	0.9–4.5
Do not know	55	17.6	13.5–22.2
Indicated as symptom of urogenital schistosomiasis			
Presence of blood in urine	284	90.7	87.0–93.7
Fever	15	4.8	2.7–7.8
Diarrhoea	9	2.9	1.3–5.4
Stomach ache	21	6.7	4.2–10.1
Itching	18	5.8	3.4–8.9
Other	1	0.3	0.0–1.8
Do not know	14	4.5	2.5–7.4
Aware of meaning of diagnosis			
Yes	225	71.9	66.5–76.8
Indicated as the way to catch the disease			
Walking barefoot	67	21.4	17.0–26.4
Eating without washing hands	38	12.1	8.7–16.3
Contact with the river	218	69.6	64.2–74.7
Drinking river water	144	46.0	40.4–51.7
Mosquito bite	11	3.5	1.8–6.2
During sexual intercourse	19	6.1	3.7–9.3
Others	5	1.6	0.5–3.7
Do not know	11	3.5	1.8–6.2
Indicated as the ‘animal’ responsible of the disease			
Mosquitoes	10	3.2	1.5–5.8
Land snail	3	1.0	0.2–2.8
River small snail	145	46.3	40.7–52.0
Fly	8	2.6	1.1–5.0
Others	5	1.6	0.5–3.7
Do not know	137	43.8	38.2–49.5
Indicated as consequences of schistosomiasis			
Anaemia	172	54.9	49.3–60.5
Smelly vaginal discharge	175	55.9	50.2–61.5
Malodorous vaginal discharge in young girls	167	53.3	47.7–59.0
Infertility	220	70.3	64.9–75.3
Cancer of the bladder	156	49.8	44.2–55.5
Death	149	47.6	42.0–53.3
Others	18	5.8	3.4–8.9

The mean (± SD) score for knowledge of schistosomiasis was 8.8 ± 2.4 out of a total of possible 13 points, yielding a 68% appropriate knowledge level for the study population. In the multivariable analysis (Additional file 4: Table S2), we found a relationship between knowledge score and history of schistosomiasis (*P* = 0.001), location (*P* = 0.005) and family size (*P* = 0.02). Indeed, a higher score level of appropriate knowledge was found for participants with a history of schistosomiasis (*α* = 0.90, 95% CI 0.35–1.45) and for those living in a household with a large number of family members (family size: 4–6;* α* = 0.86, 95% CI 0.20–1.52; family size > 6:* α* = 0.75, 95% CI 0.04–1.47). Compared to participants living in downtown Lambaréné, we found a lower score of appropriate knowledge only for participants from Zilé-PK (*α* = − 1.34, 95% CI − 2.11 to − 0.57), while we observed no difference in the score levels for participants from Mitonè-PK (*α* =  − 0.80, 95% CI − 1.89 to 0.30) and Bindo-Makouké (*α* = − 0.81, 95% CI − 1.75 to 0.14) villages,.

### Attitudes to schistosomiasis

The results on the interviewees’ attitudes toward schistosomiasis are shown in Table 5. Among the 289 children who responded to the simplified version of the questionnaire, 282 (98%; 95% CI 95–99) were ready to inform their parents if they experienced haematuria. Of the 602 participants overall, 575 (96%; 95% CI 93–97) were ready to disclose their status if they were to be found positive for schistosomiasis. Asking for the reason for disclosure, a 16 year-old boy stated that: “*if we have bilharzia, we should not be ashamed because it is a disease like any other disease*” while a 17-year-old girl stated that: “*we have to tell others so that they could help us get the drugs*”. Of the 313 young adults and adults who responded to the main questionnaire, 295 (94%; 95% CI 91–97) and 61 (19%; 95% CI 15–24) of them indicated hospitals and/or drugstores as the preferable place to seek drug treatment, respectively, while 13 (4%; 95% CI 2–7) of the participants were ready to go to the traditional healer first to seek treatment. If treated for the disease, 136 (46%; 95% CI 40–52) participants were not ready to avoid going back to the water course. In that regard, a 60-year-old woman living in a neighbourhood of Lambaréné town indicated that: “*it is the only place for me to wash and take a bath*”. With regard to the severity of the disease, 135 (45%; 95% CI 39–51) and 108 (36%; 95% CI 30–42) of the young adults and adults who responded to the main questionnaire considered schistosomiasis to be a severe or moderate disease, respectively, while 58 (19%: 95% CI 15–24) of them considered the disease as mild.

**Table 5:** Distribution of attitudes and practices to schistosomiasis among the study population

Attitudes and practices to schistosomiasis	Study population		
	*n*	%	95% CI (%)
Can disclose his/her schistosoma infectious status			
Yes	575	95.5	93.5–97.0
No	27	4.5	3.0–6.5
Can share information in case of haematuria with:^a^			
Parents	276	95.5	92.4–97.6
Friend	23	8.0	5.1–11.7
School teacher	19	6.6	4.0–10.1
Consider the gravity of schistosomiasis as:^b^			
Severe	135	44.8	39.1–50.7
Moderate	108	35.9	30.5–41.6
Mild	58	19.3	15.0–24.2
Missing data	12	–	–
Preferable source to seek medication^b^			
Hospital	295	94.2	91.1–96.6
Drugstore	61	19.5	15.2–24.3
Traditional healer	13	4.1	2.2–7.0
Will not seek the drug	0	0.0	–
Used fresh water at home			
Yes	416	69.1	65.2–72.8
No	186	30.9	27.2–34.8
Frequency of freshwater contact reported			
Every day	286	47.5	43.4–51.6
Every week	48	8.0	5.9–10.4
Sometimes a month	220	36.5	32.7–40.5
Never	48	8.0	5.9–10.4
Ready to not go to the river if being treated for schistosomiasis^b^			
Yes	136	46.3	40.4–52.1
No	158	53.7	47.9–59.5
Missing data	19	–	–
Had reported having already urinating in a watercourse			
Yes	387	64.3	60.3–68.1
No	215	35.7	31.9–40.0
Had reported having already defecating in a watercourse			
Yes	152	25.2	21.8–28.9
No	450	74.7	71.1–78.2

^a^Applicable to children only, corresponding to the simplified version of the questionnaire

^b^Applicable to young adults and adults only, corresponding to the main version of the questionnaire

The mean (± SD) score for appropriate attitudes toward schistosomiasis was 2.9 ± 0.6 points out of a total score of 4 points, giving a 73% of appropriate attitudes to schistosomiasis for the study population.

### Protective practices against schistosomiasis

Among the participants who regularly used a freshwater course for their daily activities, although some of them reported doing nothing as stated by a 56 year-old lady, some techniques are used by others to reduce the risk of being infected when going to the river particularly for a bath. A 16 year-old boy said, for instance, to (carry water from and) “*take his bath aside the river*”. In addition, a 25 year-old woman stated to “*heat the water or put it in the sun*” while a 49 year-old man said to “*put the bleach*” in the bucket of water. As another approach, a 68 year-old lady stated to “*keep clean the river all the time*” while a 16 year-old girl said to have “*stop urinating in water and avoid staying in water*”. Some inappropriate practices were reported by some participants to protect themselves to schistosomiasis such as a 63 year-old lady who said to “*must not walk without shoes*” or a 29 year old lady who reported “*to drink a lot of water*”. A 17 year-old man from his side stated that; “*I must wash myself without making too many waves*”.

### REPs toward schistosomiasis and exposure-associated risks

Practices putting respondents at risk for schistosomiasis were evaluated among the 602 study participants. Of these, 554 (92%; 95% CI 90–94) declared having been in contact with fresh water in one way or another, while 415 (69%; 95% CI 65–73) reported using freshwater from sources close to their home. As shown in Table 3, 286 (52%; 95% CI 47–56) study participants were in contact with a freshwater body every day while 48 (9%; 95% CI 6–11) and 220 (40%; 95% CI 36–44) participants were in contact with freshwater weekly or several times per month, respectively. A total of 387 (64%; 95% CI 60–68) study participants reported having urinated in a freshwater body and 152 (25%; 95% CI 22–29) reported having defecated regularly/sometimes in a freshwater body (Table 5).

The mean (± SD) score for REP was 5.4 ± 2.3 points out of a total of 9 points, indicating a 60% level of REP toward schistosomiasis in our study population. Examining factors associated with REP (Table 5), we found in multivariate analysis a relationship between the REP score level and history of schistosomiasis (*P* < 0.001), location (*P* < 0.001), the use of freshwater (*P* = 0.006) and family size (*P* = 0.007). Compared to Lambaréné town, we observed a higher score of REP for the populations of Zilé-PK (*α* = 0.77; 95% CI 0.14–1.41) and Mitonè-PK (*α* = 2.01; 95% CI 1.11–2.92) but a lower score for that of Bindo-Makouké (*α* =  − 1.36; 95% CI − 2.14 to − 0.57). We found a higher score of REP among participants with history of a diagnosis of schistosomiasis, as compared to their counterparts without such a diagnosis (*α* = 0.77; 95% CI 0.31–1.22). Similarly, we found a higher score among those who used freshwater, as compared to those who did not (*α* = 0.79; 95% CI 0.21–1.37) while, compared to families with three members or less, we found a lower score for families with four to six members (*α* =  − 0.70; 95% CI − 1.24 to − 0.15) and 7 to 30 members (*α* =  − 0.87; 95% CI − 1.47 to − 0.28) .

## Discussion

The prevalence of schistosomiasis is known to be moderate (26%) in Lambaréné itself [17] and high in the surrounding vicinity, particularly in the villages of Zilé-Pk where prevalence can reach 75% in some villages [10]. The present KAP survey, which is the first being conducted in the area, highlights a high level of population exposure to the disease due to their proximity with, and the use of, freshwater for domestic needs. The results of this study will be helpful to explain human factors pertaining to the endemicity of the disease in the region, and particularly to evaluate the role of the population behaviour for a better control of the disease [2, 18].

Treatment, either preventive or curative, and limiting human–parasite contact are key elements in schistosomiasis control. We noted that 32% of the study population had already received PZQ at some point in time. Of these, almost 50% had received the drug from CERMEL during research activities (participation in clinical studies), highlighting the impact of the research centre in the controlling the morbidity of the disease in the community. Surprisingly, only 10% of the population indicated having received PZQ from MDA national campaigns, bringing into question both the coverage and impact of these campaigns on regional disease control. Indeed, as we previously indicated, only one national campaign of MDA of PZQ was conducted among school children during the last decade.

The present survey revealed that almost the entire study population had already heard of schistosomiasis, leading to the assumption that the local population is well aware of the presence of the disease in the community. This situation of high awareness corroborates observations made in other areas endemic for schistosomiasis [19, 20]. In our population, the main avenues of information on the disease was by word of mouth (57%) and at school (30%).

Overall, we found that the local population had a fairly good knowledge level of the disease. However, compared to the other study areas, the level of correct knowledge of schistosomiasis found for the Zilé-Pk villages, known for their high prevalence of schistosomiasis, was low, similar to data reported from some other areas of Africa. Indeed, a low disease awareness level was reported in western Kenya despite the observation of high disease prevalence [21]. Based on our results, although almost the entire population was aware of the presence of the disease, we can assume that some aspects of the disease remain unclear, particularly for the population of Zilé-PK. It has been reported that having heard about the disease is not necessarily associated with correct knowledge on how the disease is acquired, transmitted, or prevented [8, 20, 22]. In northern Senegal, for example, where 86% of the population had heard about schistosomiasis, only 30% had adequate knowledge of its mode of transmission [23]. Up to 91% of our study population indicated haematuria as the main symptom of the disease and up to 30% failed to indicate knowledge of the connection between a freshwater body, and catching the disease. Only 46% indicated river small snails as animals being involved in transmitting the disease in a context where providing safe water is difficult, indicating the necessity of educating people on disease transmission, for example at school or through the media. Indeed, health education for schistosomiasis has been shown to considerably improve the rate exposure to infested water and the infection rate in a population living in a heavily endemic area with wells as drinking water sources [4].

We found the overall attitude level of our population on schistosomiasis to be good. We noted, for example, that people were open to talking about the disease, which could explain our finding of a positive association between a higher level of knowledge and a higher number of persons living in the same household. Indeed, all children were ready to inform their parents in case of visible blood in urine, and nine out of ten young adults and adults were ready to share their status with others if they were ever found to be infected. It appears that the local population considers schistosomiasis to be like any other disease and therefore think that there is no need to be ashamed when having it; consequently, the disease does not seem to be associated with stigma. By sharing their infectious status, they expect in fact to be advised on how to proceed to find a solution. This situation highlights the predisposition of the population to seek treatment if infected. Interestingly, almost all participants preferred to go either directly to a hospital or drugstore to obtain drugs for treatment, rather than, for example, consult a traditional healer first. We conclude that if PZQ is available and affordable in the area, it will be used by large numbers of the afflicted population. This is in contrast to some observations in the field where some people have been found to be reluctant to participate in an MDA campaign, particularly whilst being asymptomatic; however, this is not specific to the MDA of PZQ.

The population using freshwater for their daily activities, particularly at home, reported some actions taken to protect themselves from the disease. Although these methods need to demonstrate their efficiency, at least it assumes that the population are ready to follow recommended preventive measures, as far as living circumstances permit. We can therefore suppose that education of the population on protective measures to the disease can be successfully implemented in the local population and could further contribute to improving the situation. In addition to door-to-door campaigns, sending messages on protective measures through social networks or billboards at the main human–water contact points could alleviate schistosomiasis transmission in the community.

Two main points were considered in the assessment of the level of population REP to schistosomiasis, namely freshwater contact and urinating/defaecating into a water body. The results of our questionnaire show a rather high level of REP in the population. In an area where around 70% of the population lives close to a water course and where 71% use fresh water at home as the water source for their daily activities, this finding is not surprising, but rather confirms the fact that some parts of the population are living in conditions putting them almost inevitably in contact with the parasite. We can assume that this is particularly true for the areas surrounding the city of Lambaréné, such as the Mitonè-PK area, where no tap water is available, and, to a lesser extent, the villages of Zilé-PK. Compared with the urban setting of Lambaréné, we found that the level of REP is higher in the Zilé-PK area and Mitonè-PK areas, and lower in the villages of Bindo-Makouké where tap water or public water pumps are available for the majority of the population. We can therefore assume that the high prevalence of schistosomiasis observed for some parts of Lambaréné city [17] is particularly due to the neighbourhoods lacking an adequate water supply network. In order to interrupt the life cycle of the parasite, the most important action to undertake is to provide the population with safe water. Unfortunately, this remains difficult to achieve, at least in the short- or medium-term. In the absence of alternatives to open freshwater use, it is essential to identify the most exposed population groups, as health education measures could then focus on how to adapt their behaviour in order to reduce their level of exposure to the disease.

The absence of safe water cannot solely explain the high schistosomiasis endemicity in the region. One key element in the transmission of the disease is the contamination of a freshwater body by infected individuals. We were therefore interested to know how local population could infest the watercourse. Our study reveals that up to 64% of the population stated having urinated in the watercourses, which makes clear that the habit of urinating in watercourses plays a major role in the presence of the disease. Focusing the education of the population on that simple practice could thus contribute to considerably alleviating the burden of the disease in the locality.

Knowledge as well as REP levels both increased with an individual’s past medical history of schistosomiasis. This result supports the notion that the population practices with regard to schistosomiasis are less clearly linked to their knowledge level on the disease, but rather to their living conditions. Indeed, more than half of the population, for well-understood reasons, is not ready to avoid freshwater contact in order not to get infected, or become re-infected, even if requiring treatment for schistosomiasis. Instead of not being aware of the risk to be infected or re-infected, it appears that this attitude is mainly due to the fact that these individuals simply do not have any other water source than stream freshwater for their daily activities. We also noted that the number of household members can influence the practices regarding schistosomiasis. Families with many members perform fewer REP per capita, probably because only some members of the family are assigned to particular household chores. We can imagine that these are the women and/or the children; however, our results did not shown any difference in term of REP level according to age and gender. Most likely, people with a high level of REP can be found among those living closest to a watercourse, particularly in areas where pipe water is not available, or scarce. To be efficient, implementation of schistosomiasis control programme should thus focus on this particular population group.

## Conclusions

The level of population exposure to schistosomiasis in Lambaréné and particularly in the surrounding rural areas of the city is high. The present KAP survey reveals a fairly good knowledge level and a good attitude level of the population toward schistosomiasis, but also a high level of REPs, assumed to be due to their living conditions. In the context of this study, carried out in areas where it is difficult to provide safe water for some of the population, the implementation of the WHO schistosomiasis control programme should focus on two mains aspects, namely the effectiveness of PZQ treatment and education of at-risk populations on preventive behaviours as it is in these populations that a lack of knowledge on the disease transmission was observed. Populations with known freshwater contact could, for instance, be encouraged to seek regular treatment at least once per year, in addition to the implementation of measures aimed at reducing their exposure to the disease.

## Supplementary Information

**Additional file 1:Text S1.** Questionnaire for adults who participated in the study.

**Additional file 2:Text S2.** Questionnaire for children who participated in the study.

**Additional file 3:Table S1.** Description of the scoring of each question and total score for knowledge, attitudes and practices calculated for the participants interviewed during the surveys.

**Additional file 4:Table S2.** Factors associated with appropriate knowledge and risk-enhancing practices of the study population towards schistosomiasis.

## Data Availability

The datasets used and analysed during the current study are available from the corresponding author on reasonable request.

## Abbreviations

CEI: Comité d’Ethique Institutionel
CERMEL: Centre de Recherches Médicales de Lambaréné
CI: Confidence interval
KAP: Knowledge, attitude and practices
MDA: Mass drug administration
NTD: Neglected tropical disease
PZQ: Praziquantel
REP: Risk-enhancing practices
RN1: Route nationale 1
SD: Standard deviation
WASH: WAter Sanitation and Hygiene
WHO: World Health Organization
