# Supplementary material for: Knowledge, attitudes and practices pertaining to urogenital schistosomiasis in Lambaréné and surrounding areas, Gabon
Source: Parasit Vectors. 2021 Sep 22;14:486. doi: 10.1186/s13071-021-04905-0 (PMC8456596; doi:10.1186/s13071-021-04905-0)
Supplement: Supplementary file 1 — Additional file 1:Text S1. Questionnaire for adults who participated in the study. [file 13071_2021_4905_MOESM1_ESM.pdf]

Centre de Recherches Médicales de Lambaréné

www.cermel.org
BP 242 · Lambaréné
BP 1437 · Libreville
Gabon
Tel: +241 07 98 91 91
admin@cermel.org

Numéro de participation à l'étude : SK-|__ |__ |__ | **Initiales** | __ | __ | __ | __ |

Schistosomiase: Connaissance, Attitude et Pratiques des Populations de Lambaréné et ses Environs

Acronyme: SchistoKAP

Version 1.3_16.06.2019

Questionnaire Adulte

CONSENTEMENT VOLONTAIRE A PARTICIPER A L'ETUDE

S'il s'agit d'un participant âgé de 18 ans et plus, a-t-il signé le formulaire de consentement éclairé ?

1. ☐ Oui, 2. ☐ Non

S'il s'agit d'un volontaire âgé de moins de 18 ans ;

Le volontaire a-t-il donné son assentiment oral de participer à l'étude ?

1. ☐ Oui, 2. ☐ Non

Le parent ou tuteur responsable légal a-t-il signé le formulaire de consentement éclairé ?

1. ☐ Oui, 2. ☐ Non

Q1. IDENTIFICATION DU PARTICIPANT ET DONNEES SOCIODEMOGRAPHIQUES

Démographie

Q1.1-Quel est votre âge ? |__ |__ | ans

Q1.2-Quelle est votre date de naissance ?

|__ |__ | |__ |__ |__ | |__ |__ |__ |__ |
Jour Mois Année

Q1.3 Vous êtes ?

1. ☐ Un homme, 2. ☐ Une femme

Q1.4-Dans quel quartier habitez-vous? _

Q1.5-Avez-vous toujours vécu dans ce quartier/village?

1. ☐ Oui, 2. ☐ Non

Q1.6-Si "Non", quand êtes-vous arrivé ici? | __ | __ | | __ | __ | __ | |__ |__ |__ |__ |
Jour Mois Année

Q1.7-Si "Non", quel quartier/ville habitiez-vous auparavant? _

1. ☐ En zone rurale, 2 ☐ En zone urbaine, 3 ☐ En zone semi-urbaine

Données socio-économiques

Q1.8-Quel est votre niveau scolaire?

1. ☐ Aucun, 2 ☐ Primaire, 3 ☐ Secondaire, 4 ☐ Université

Q1.9-Quelle est votre occupation principale?

1. ☐ Aucun, 2. ☐ Agriculteur, 3. ☐ Pêcheur,
4. ☐ Employé de bureau, 5. ☐ Commerçant, 6. ☐ Étudiant,
7. ☐ Agent de santé, 8. ☐ Autre, _

Q1.10-Le revenu mensuel de votre ménage peut-être estimé à :

1. ☐ Non applicable, 2. ☐ <50 000Fcfa, 3. ☐ 50 000-150 000Fcfa,
4. ☐ Non applicable, 5. ☐ <50 000Fcfa, 6. ☐ Ne veut pas répondre

Conditions de vie

Q1.11-Quelle est la principale source d'eau pour vos activités quotidiennes à la maison?
1. ☐ SEEG, 2. ☐ Rivière, 3. ☐ Puits,
4. ☐ Autre, précisez: _

Q1.12-Estimez-vous que votre maison est située à proximité d'un point d'eau: lac, rivière, ruisseau, marais?
1. ☐ Oui, 2. ☐ Non

Q1.13-Si "Oui", veuillez spécifier le type de cours d'eau
1. ☐ Lac, 2. ☐ Rivière, 3. ☐ Ruisseau, 4. ☐ Marais

Q1.14–Quelle est la source d'eau pour vos activités quotidiennes à l'école ou au lieu de travail?
1. ☐ SEEG, 2. ☐ Rivière, 3. ☐ Puits,
5. ☐ Autre précisez: _

Q1.15-Estimez-vous que votre école ou lieu de travail est situé à proximité d'un cours d'eau: lac, rivière, ruisseau, marais?
1. ☐ Oui, 2. ☐ Non

Q1.16- Si "Oui", veuillez spécifier le type de cours d'eau
1. ☐ Lac, 2. ☐ Rivière, 3. ☐ Ruisseau, 4. ☐ Marais

Q1.17–Combien de personnes êtes-vous dans votre maison? _ _ _ _ _ _ _ personnes

Environnement sanitaire

Q1.18-Quel type de sanitaire (toilettes, latrine) utilisez-vous chez vous?
1. ☐ Interne (privé), 2. ☐ Latrine commune, 3. ☐ Latrine traditionnelle
4. ☐ Dans la broussaille, 5. ☐ Nous n'avons pas de latrine dans notre maison

Q1.19-Quel type de sanitaire (toilettes, latrine) utilisez-vous à l'école ou à votre lieu de travail?
1. ☐ Interne (privé), 2. ☐ Latrine commune, 3. ☐ Latrine traditionnelle
4. ☐ Dans la broussaille, 5. ☐ Il n'y a pas de latrine dans mon école / lieu de travail

Q2. ÉVALUATION DE LA CONNAISSANCE SUR LA BILHARZIOSE

Connaissance sur la définition de la schistosomiase

Q2.1-Avez-vous déjà entendu parler de la bilharziose

1. ☐ Oui, pouvez-vous nous dire en termes simples ce qu'est la bilharziose? _ _ _ _ _ _ _ _
_ _
2. ☐ Non (=> *Allez à la question Q3.5*)

Q2.2-Si "Oui", où avez-vous entendu parler de la bilharziose pour la première fois?

1. ☐ À l'école
2. ☐ À travers un parent, un ami ou un collègue (bouche à oreille)
3. ☐ À la télévision ou à la radio
4. ☐ À travers un prospectus
5. ☐ Lors d'une campagne de santé
6. ☐ À l'hôpital
7. ☐ À travers le laboratoire de recherche (CERMEL)
8. ☐ Autre: _

Q2.3-Si "Oui", connaissez-vous un nom local donné à la bilharziose?

1. ☐ Oui, lequel: _
2. ☐ Non

Q2.4-Si "Oui", pensez-vous que la bilharziose est une maladie?

1. ☐ Oui, 2. ☐ Non, 3. ☐ Je ne sais pas

Q2.5-Si vous pensez que la bilharziose est une maladie, pensez-vous que c'est une maladie grave?

1. ☐ Oui, 2. ☐ Non

Q2.6-Si "Oui", quel est l'agent causal de la bilharziose (le germe responsable de la bilharzie)?

1. ☐ Un virus
2. ☐ Un ver
3. ☐ Une bactérie
4. ☐ Autre : _
5. ☐ Je ne sais pas

Connaissance sur le diagnostic de la schistosomiase

Q2.7-Pour vous, quels sont les signes qui montrent que quelqu'un est atteint de bilharziose?

1.-_ _
2.-_ _
3. ☐ Je ne sais pas

Q2.8-Selon vous, comment savez-vous que quelqu'un est atteint de bilharziose?

1. ☐ Quand il a de la fièvre
2. ☐ Quand il fait la diarrhée
3. ☐ Quand il a mal au ventre
4. ☐ Quand il pisse du sang
5. ☐ Quand il a des démangeaisons
6. ☐ Je ne sais pas
7. ☐ Autre: _
_ _

Q2.9-Savez-vous s'il existe un test à l'hôpital pour rechercher la bilharziose chez un individu?

1. ☐ Oui, 2. ☐ Non

Si oui, lequel? _

Connaissance sur la prévention de la schistosomiase
**Q2.10-Comment une personne peut-elle se protéger de la bilharziose?**<br>1. ☐ Eviter d'uriner dans la rivière<br>2. ☐ Éviter de faire des selles dans la rivière<br>3. ☐ Eviter d'aller à la rivière<br>4. ☐ Éviter de marcher pieds nus<br>5. ☐ Dormir sous la moustiquaire<br>6. ☐ Autre, pouvez-vous donner des détails svp: _<br>_ _
Connaissance sur la transmission de la schistosomiase
**Q2.11-Comment peut-on contracter la bilharziose?**<br>1. ☐ En Marchant pieds nus<br>2. ☐ En mangeant sans se laver les mains<br>3. ☐ En allant à la rivière<br>4. ☐ En buvant l'eau de la rivière<br>5. ☐ Quand on est piqué par les moustiques<br>6. ☐ Pendant les rapports sexuels avec une personne atteinte de bilharziose<br>7. ☐ Je ne sais pas<br>8. ☐ Autre, spécifiez _<br><br>**Q2.12-Pensez-vous que la bilharziose est contagieuse?**<br>1. ☐ Oui,<br>2. ☐ Non<br>3. ☐ Voulez-vous commenter votre réponse? _<br>_ _<br><br>**Q2.13-Connaissez-vous l'animal qui transmet la bilharziose?**<br>1. ☐ Je ne sais pas<br>2. ☐ Le moustique<br>3. ☐ Le grand escargot terrestre<br>4. ☐ Le petit escargot de rivière<br>5. ☐ La mouche<br>6. ☐ Autre, précisez _
Connaissance sur traitement de la schistosomiase
**Q2.14-Pensez-vous que la bilharziose peut guérir sans traitement?**<br>1. ☐ Oui,   2. ☐ Non<br><br>**Q2.15-Pensez-vous qu'il existe un médicament pour traiter la bilharziose?**<br>1. ☐ Oui,   2. ☐ Non<br><br>**Q2.16-Connaissez-vous un traitement traditionnel de la bilharziose?**<br>1. ☐ Oui,   2. ☐ Non<br><br>**Q2.17-Si oui, pensez-vous que ce traitement traditionnel est efficace, que ça traite vraiment?**<br>1. ☐ Oui,   2. ☐ Non<br><br>**Q2.18-Selon vous, comment pouvez-vous savoir qu'une personne est guérie de la bilharziose?** _<br>_ _<br>_ _<br><br>**Q2.19-Avez-vous des commentaires sur le traitement de la bilharziose?** _ _ _ _ _ _ _ _ _ _ _ _ _<br>_ _<br>_ _

Q3. EVALUATION DE L'ATTITUDE DE LA POPULATION SUR LA BILHARZIE

Perception de la maladie

Q3.1-Pensez-vous que nous devrions avoir honte d'avoir la bilharziose?

1. ☐ Oui,
2. ☐ Non,
3. ☐ Pouvez-vous expliquer votre réponse? _
_ _
_ _

Q3.2-Selon vous, la bilharziose est une maladie qui attaque:

1. ☐ Les enfants
2. ☐ Les femmes
3. ☐ Les hommes
4. ☐ Les pauvres
5. ☐ Les gens riches
6. ☐ Les personnes faibles
7. ☐ Avez-vous un commentaire sur cette question? _
_ _

Q3.3-Pensez-vous que la bilharziose peut causer?

1. ☐ L'anémie
2. ☐ La stérilité (infertilité)
3. ☐ Le cancer de la vessie
4. ☐ Les pertes vaginales malodorantes chez les femmes
5. ☐ Les pertes vaginales malodorantes chez la petite fille
6. ☐ La mort
7. ☐ Autre, précisez: _
_ _

Q3.4-A quel niveau de gravité classez-vous la bilharziose? (Encerclez une réponse)

0	1	2	3	4	5
(Pas grave du tout)					(Très grave)

Q3.5-Si vous pensez que vous avez la bilharziose, iriez-vous chercher des soins:

1. ☐ À l'hôpital**?**
2. ☐ À la pharmacie?
3. ☐ Chez un guérisseur traditionnel?
4. ☐ Personne, je n'irai pas consulter
5. ☐ Avez-vous un autre commentaire sur cette question? _ _ _ _ _ _ _ _ _ _ _ _ _ _ _ _ _ _ _
_ _
_ _

Q3.6-Informeriez-vous vos proches si vous avez la bilharziose?

1. ☐ Oui,
2. ☐ Non,
3. ☐ Pouvez-vous argumenter votre réponse? _
_ _
_ _

Q4. ÉVALUATION DES PRATIQUES PAR RAPPORT À LA BILHARZIE

Pratiques relatives à l'exposition à la bilharziose

Q4.1-Avez-vous été en contact avec un cours d'eau (lac, rivière, ruisseau, marais, etc.)?
1. ☐ Oui, 2. ☐ Non *(=> Allez à la question Q5.1)*

Q4.2-Si "Oui", de quel type de cours d'eau s'est-il agit ?
1. ☐ Lac, 2. ☐ Rivière, 3. ☐ Ruisseau, 4. ☐Autre ……………….……….

Q4.3-Si "Oui", où avez-vous été en contact avec le cours d'eau?
1. ☐ À la maison, 2. ☐ À l'école /lieu de travail, 3. ☐ À la plantation
4. ☐ Autre : _

Q4.4-Si "Oui", à quelle fréquence?
1. ☐ Tous les jours, 2. ☐ Toutes les semaines, 3. ☐ Quelques fois par mois

Q4.5-Si "Oui", quand y êtes-vous allé pour la dernière fois? | __ | __ | | __ | __ | __ | 20 __ | __ |
Jour mois Année

Q4.6-Si "Oui", quelles sont les raisons pour lesquelles vous avez été (ou vous êtes) en contact avec le cours d'eau?
1. ☐ Pour les travaux ménagers (vaisselle, lessive…)
2. ☐ Pour puiser de l'eau
3. ☐ Pour prendre un bain
4. ☐ Pour jouer
5. ☐ Pour pêcher
6. ☐ Pour mes activités agricoles
7. ☐ Autre, précisez: _

Q4.7-Si "Oui", à quelle heure de la journée partez-vous habituellement au cours d'eau?
1. ☐ Le matin
2. ☐ Entre 12 heures et 15 heures
3. ☐ Dans l'après-midi, à partir de 15 heures à 18 heures
4. ☐ Le soir ou la nuit
5. ☐ Je n'ai pas d'heures précises pour aller à la rivière

Q4.8- Si "Oui", avez-vous déjà uriné dans ce (ou un autre) cours d'eau?
1. ☐ Oui, 2. ☐ Non

Q4.9- Si "Oui", avez-vous déjà fait les selles dans ce (ou un autre) cours d'eau?
1. ☐ Oui, 2. ☐ Non

Pratiques relatives à la protection contre la schistosomiase

Q4.10-Quelle est votre pratique quotidienne pour éviter d'avoir la bilharziose? _ _ _ _ _ _ _ _
_ _
_ _

Pratiques concernant le traitement de la schistosomiase

Q4.11-Si vous êtes traité pour la bilharziose, pourriez-vous éviter d'aller à la rivière pour ne pas avoir à nouveau la bilharziose?
1. ☐ Oui
2. ☐ Non
3. ☐ Pouvez-vous argumenter votre réponse? _
_ _

Q5. EVALUATION CLINIQUE

Q5.1-Avez-vous déjà eu du sang dans les selles?
1. ☐ Oui, 2. ☐ Non

Q5.2-Avez-vous déjà uriné avec du sang?
1. ☐ Oui, 2. ☐ Non (=> *Allez à la question Q5.4*)

Q5.3-Si "Oui", quand avez-vous uriné avec du sang la dernière fois?
1. ☐ Cette semaine
2. ☐ Ce mois-ci
3. ☐ Le mois dernier
4. ☐ Pendant l'année
5. ☐ Plus d'un an
6. ☐ Je ne me rappelle pas

Q5.4-Avez-vous déjà eu la bilharziose?
1. ☐ Oui, pouvez-vous dire quand? _ _ _ _ _ (jour) _ _ _ _ _ _ _ (mois) _ _ _ _ _ _ (années)
2. ☐ Non (=> *Allez à la question Q5.6*)

Q5.5-Si "Oui", comment avez-vous su que vous aviez la bilharziose? _ _ _ _ _ _ _ _ _ _ _ _ _ _ _
_ _

Q5.6-Avez-vous déjà reçu le médicament de la bilharziose?
(Présentez une copie du médicament au volontaire, s'il vous plaît)
1. ☐ Oui, 2. ☐ Non (=> *Allez à la question Q5.9*)

Q5.7-Si "Oui", vous souvenez-vous quand? _ _ _ _ (jour) _ _ _ _ _ _ _ (mois) _ _ _ _ _ _ (années)

Q5.8-Si "Oui", comment êtes-vous entré en possession du médicament?
1. ☐ Je l'ai acheté à la pharmacie
2. ☐ Je l'ai reçu du laboratoire de recherche (CERMEL)
3. ☐ Je l'ai reçu lors de campagnes de traitement de masse (programme national)
4. ☐ Je l'ai reçu dans un centre de santé / à l'hôpital
5. ☐ Je l'ai reçu d'un parent
6. ☐ Autre, précisez: _
_ _

Q5.9-Que pensez-vous du médicament de la bilharziose? _
_ _
_ _

Merci d'avoir pris le temps de répondre à ce questionnaire. Nous espérons que les informations que vous nous avez fournies contribueront à un meilleur contrôle de la bilharziose dans notre localité.

L'équipe de recherche

Date du jour : |__|__| |__|__|__| 2019 Initiales intervieweur : |__|__|__|
