# Supplementary material for: Knowledge, attitudes and practices pertaining to urogenital schistosomiasis in Lambaréné and surrounding areas, Gabon
Source: Parasit Vectors. 2021 Sep 22;14:486. doi: 10.1186/s13071-021-04905-0 (PMC8456596; doi:10.1186/s13071-021-04905-0)
Supplement: Supplementary file 2 — Additional file 2:Text S2. Questionnaire for children who participated in the study. [file 13071_2021_4905_MOESM2_ESM.pdf]

Centre de Recherches Médicales de Lambaréné

www.cermel.org
BP 242 · Lambaréné
BP 1437 · Libreville
Gabon
Tel: +241 07 98 91 91
admin@cermel.org

Numéro de participation à l'Etude: SK-| __ | __ | __ | **Initiales | __ | __ | __ | __ |**

Schistosomiase : Connaissance, Attitude et Pratiques des populations de Lambaréné et de ses Environs

Acronyme : SchistoKAP

Version 1.2_04.06.2019

<u>Questionnaire Enfant</u>

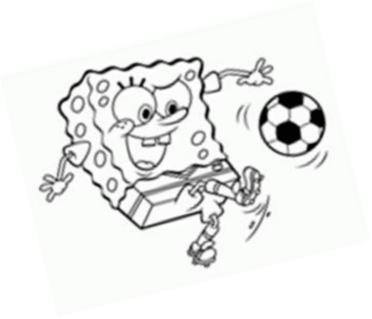

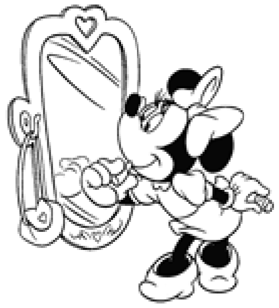

SK -| __ | __ | __ |

Consentement volontaire à participer à l'étude
**Le volontaire a-t-il donné son accord oral de participer à l'étude ?**<br>1. ☐ Oui, 2. ☐ Non (Ne pas procéder au questionnaire svp)<br><br>**Le parent ou tuteur responsable l'enfant a-t-il signé le formulaire de consentement éclairé ?**<br>1. ☐ Oui, 2. ☐ Non (Ne pas procéder au questionnaire svp)

Q1. Identification et données sociodémographiques

Données démographiques

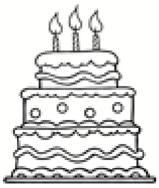

Q1.1- Quel âge as-tu ? __|__| ans

Q1.2- Quand est-ce que tu fêtes ton anniversaire ? |__|__| |__|__|__| |__|__|__|__|

Q1.3- Es-tu ;

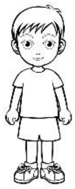 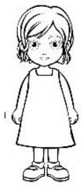

1. ☐ Un garçon ? 2. ☐ Une fille ?

Q1.4- Quel est le nom de ton quartier ? _

Q1.7- Quel quartier habitais-tu avant ? _

1. ☐ Zone rurale 2. ☐ Zone urbaine 3. ☐ Zone semi-urbaine

Données socio-économiques

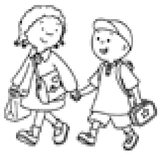

Q1.8- Où vas-tu à l'école ?
1. ☐ Je ne vais pas à l'école 2. ☐ A l'école primaire 3. ☐ Au lycée

Q1.8a- Si tu vas à l'école, comment s'appelle ton école ? _

Q1.8b- Et quelle classe fais-tu ? _

Conditions de vie

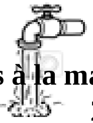

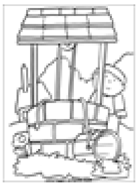

Q1.11- Quelle eau utilisez-vous à la maison ?
1. ☐ L'eau de la SEEG 2. ☐ L'eau de la rivière 3. ☐ L'eau du puit

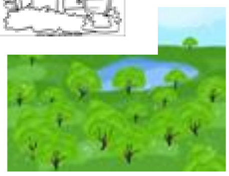

Q1.12- Penses-tu que votre maison est proche d'un point d'eau ?
1. ☐ Oui 2. ☐ Non

Q1.13- Si oui, s'agit-il :
1. ☐ D'un lac? 2. ☐ D'une rivière ? 3. ☐ D'un marigot ?

Q1.14- Quelle eau utilises-tu à l'école ?
1. ☐ L'eau de la SEEG 2. ☐ L'eau de la rivière 3. ☐ L'eau du puit

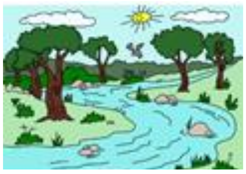

Q1.15- Penses-tu que ton école est proche d'un point d'eau ?
1. ☐ Oui 2. ☐ Non

Q1.16- Si oui, s'agit-il :
1. ☐ D'un lac ? 2. ☐ D'une rivière? 3. ☐ D'un marigot ?

Environnement sanitaire

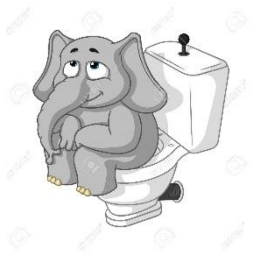 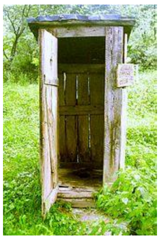 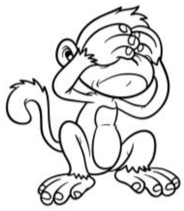

Q1.18- Quel type de toilettes utilisez-vous à la maison ?

1. ☐ Moderne 3. ☐ Traditionnelles 5. ☐ Nous n'avons pas de toilette à la maison

Q1.19- Quel type de toilettes utilises-tu à l'école ?

1. ☐ Moderne 3. ☐ Traditionnelles 5. ☐ Nous n'avons pas de toilette à l'école

Q2. Evaluation de la connaissance sur la Bilharzie

Q2.1- As-tu déjà entendu parler de la bilharzie ?

1. ☐ Oui 2. ☐ Non (=> *Allez à la question Q3.6*)

Q2.2- Si oui, où as-tu entendu parler de la bilharzie pour la première fois ?

1. ☐ A l'école
2. ☐ A la maison (Parent, famille)
8. ☐ Autre, précisez _

Q2.20- Si oui, sais-tu quand quelqu'un est malade de la bilharzie ?

1. ☐ Oui, comment ? _
2. ☐ Non

Q2.21- Si oui, sais-tu où est ce qu'on attrape la bilharzie ?

1. ☐ Oui, où ? _
2. ☐ Non

Q2.22- Si oui, que peux-tu faire pour éviter la bilharzie ? _

_ _

Q3. Evaluation de l'attitude vis-à-vis de la bilharzie

Q3.6- S'il t'arrive de pisser du sang, le diras-tu aux autres?

1. ☐ Oui 2. ☐ Non

Q3.6a- S'il t'arrive de pisser du sang, à qui le diras-tu ?

1. ☐ A mes parents
2. ☐ A mon enseignant (e)
3. ☐ A mes amis

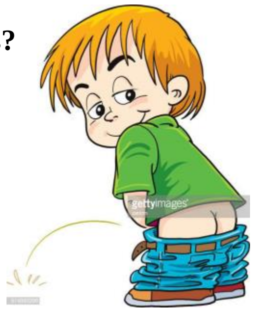

Q4. Evaluation des pratiques vis-à-vis de la bilharzie

Q4.1- Vas-tu souvent à l'eau ?

1. ☐ Oui 2. ☐ Non (=> *Allez à la question Q5.1*)

Q4.2- Si oui, s'agit-il :

1. ☐ D'un lac ? 2. ☐ D'une rivière ? 3. ☐ D'un marigot ?

Q4.3- Si oui, d'où est-ce que tu pars le plus souvent pour le point d'eau ?

1. ☐ De la maison 2. ☐ A l'école 4. ☐ Autre, _

Q4.4- Si oui, combien de fois vas-tu au point d'eau ?

1. ☐ Tous les jours 2. ☐ Parfois (De temps en temps)

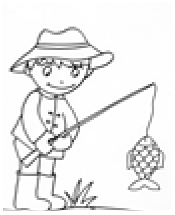

Q4.6- Si oui, pourquoi vas-tu à ce point d'eau ?

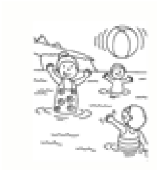

1. ☐ Pour laver le linge ou faire la vaisselle
2. ☐ Pour puiser de l'eau
3. ☐ Pour me baigner
4. ☐ Pour jouer avec mes amis
5. ☐ Pour la pèche
6. ☐ Pendant les travaux champêtres

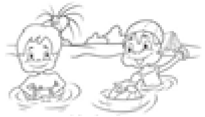

Q4.7- Si oui, à quel moment de la journée tu y vas le plus souvent ?

1. ☐ En matinée 3. ☐ En après-midi 5. ☐ Je n'ai pas d'heure pour aller à la rivière

Q4.8- Si oui, as-tu déjà uriné dans ce point d'eau quand tu y vas ?

1. ☐ Oui 2. ☐ Non

Q4.9- Si oui, as-tu déjà fait caca dans ce point d'eau quand tu y vas ?

1. ☐ Oui 2. ☐ Non

Q5. Clinique

Q5.1- As-tu déjà cabiné du sang ?

1. ☐ Oui 2. ☐ Non

Q5.2- As-tu déjà pissé du sang ?

1. ☐ Oui 2. ☐ Non

Q5.4- On t'a déjà dit que tu as la bilharzie ?

1. ☐ Oui 2. ☐ Non (=> *Allez à la question Q5.6*)

Q5.5- Si oui, qui te l'a dit ?

1. ☐ Mes parents 2. ☐ A l'hôpital 3. ☐ Mes amis 4. ☐ Autre, _ _ _ _ _

Q5.6- As-tu déjà recu le médicament de la Bilharzie ?

1. ☐ Oui 2. ☐ Non

Q5.8- Si oui, où as-tu recu ce médicament ?

2. ☐ Au CERMEL 3. ☐ A l'école 4. ☐ A l'hôpital 5. ☐ A la maison par mes parents

Nous te remercions pour ta participation à cette étude. Les réponses que tu viens de nous donner vont nous aider à mieux réfléchir sur comment te protéger, toi, ta famille et tes amis contre la bilharzie.

L'équipe de recherche

Date du jour : |__|__| |__|__|__| 2019 Initiales intervieweur |__|__|__|
