# Supplementary material for: Knowledge, attitudes and practices pertaining to urogenital schistosomiasis in Lambaréné and surrounding areas, Gabon
Source: Parasit Vectors. 2021 Sep 22;14:486. doi: 10.1186/s13071-021-04905-0 (PMC8456596; doi:10.1186/s13071-021-04905-0)
Supplement: Supplementary file 3 — Additional file 3:Table S1. Description of the scoring of each question and total score for knowledge, attitudes and practices calculated for the participants interviewed during the surveys. [file 13071_2021_4905_MOESM3_ESM.pdf]

Knowledge, attitudes and practices pertaining to urogenital schistosomiasis in Lambaréné and surrounding areas, Gabon

Table S1: Description of the scoring of each question and total score for knowledge, attitudes and practices calculated for the participants interviewed during the surveys

Question reference number[$]	Answer	Mark per answer	Maximum score for	
			Adults	Children
**Knowledge**				
Q2.1	Oui	+1	1	1
	Non	+0		
Q2.4	Oui	+1	1	NA
	Non	+0		
Q2.6	Un virus	0+	1	NA
	Un ver	+1		
	Une bactérie	+0		
	Autre	+0		
	Je ne sais pas	+0		
Q2.8	Quand il a de la fièvre	+0	1	NA
	Quand il fait la diarrhée	+0		
	Quand il a mal au ventre	+0		
	Quand il pisse du sang	+1		
	Quand il a des démangeaisons	+0		
	Je ne sais pas	+0		
	Autre	+0		
Q2.9	Oui	+1	1	NA
	Non	+0		
Q2.10	Eviter d'uriner dans la rivière	+1	3	NA
	Eviter de faire des selles dans la rivière	+1		
	Eviter d'aller à la rivière	+1		
	Eviter de marcher pieds nus	+0		
	Dormir sous la moustiquaire	+0		
	Autre	+0		
Q2.11	En marchant pieds nus	+0	1	NA
	En mangeant sans se laver les mains	+0		
	En allant à la rivière	+1		
	En buvant l'eau de la rivière	+0		
	Quand on est piqué par les moustiques	+0		
	Pendant les rapports sexuels avec une personne atteinte de la bilharzie	+0		
	Je ne sais pas	+0		
	Autre	+0		
Q2.12	Oui	+0	1	NA
	Non	+1		
Q2.13	Je ne sais pas	+0	1	NA
	Le moustique	+0		
	Le grand escargot terrestre	+0		
	Le petit escargot de rivière	+1		
	La mouche	+0		
	Autre	+0		
Q2.14	Oui	+0	1	NA
	Non	+1		
Q2.15	Oui	+1	1	NA
	Non	+0		
**Total score for knowledge**			**13**	**1**

**Attitudes**				
Q3.1	Oui	+0	1	NA
	Non	+1		
Q3.5	A l'hôpital ?	+1	2	NA
	A la pharmacie ?	+1		
	Chez un guérisseur traditionnel ?	+0		
	Personne, je n'irai pas consulter	+0		
Q3.6	Oui	+1	1	1
	Non	+0		
**Total score for attitudes**			**4**	**1**

**Practices**				
Q4.1	Oui	+1	1	1
	Non	+0		
Q4.4	Tous les jours	+3	3	3
	Toutes les semaines	+2		
	Quelques fois par mois	+1		
Q4.7	Le matin	+0	1	1
	Entre 12 heures et 15 heures	+1		
	Dans l'après-midi, à partir de 15 heures à 18 heures	+1		
	Le soir ou la nuit	+0		
	Je n'ai pas d'heure précises pour aller à la rivière	+1		
Q4.8	Oui	+2	2	2
	Non	+0		
Q4.9	Oui	+1	1	1
	Non	+0		
Q4.11	Oui	+1	1	NA
	Non	+0		
Children sub-total score for risk enhanced practices			-	8
**Total score for risk enhanced practices**			**9**	**9***

$^{\$}$Reference number of the question in the adults and children questionnaires

*To harmonise the score between adults and children particularly for multivariate analysis, the total score of children was obtained by multiplying the sub-total score by eight and divided by nine

NA: Not Applicable
