# Supplementary material for: Knowledge, attitudes and practices pertaining to urogenital schistosomiasis in Lambaréné and surrounding areas, Gabon
Source: Parasit Vectors. 2021 Sep 22;14:486. doi: 10.1186/s13071-021-04905-0 (PMC8456596; doi:10.1186/s13071-021-04905-0)
Supplement: Supplementary file 4 — Additional file 4:Table S2. Factors associated with appropriate knowledge and risk-enhancing practices of the study population towards schistosomiasis. [file 13071_2021_4905_MOESM4_ESM.pdf]

Additional File 4 – Table S2. Factors associated with appropriate knowledge and risk-enhancing practices of the study population towards schistosomiasis.

**Variable**	**Correct knowledge score***							**Risk-enhancing practices score**						
	Mean	Unadjusted analysis			Adjusted analysis[β]			Mean	Unadjusted analysis			Adjusted analysis		
		α	95%CI(α)	*p*	α	95%CI(α)	*p*		α	95%CI(α)	*p*	α	95%CI(α)	*p*
**Age**				**0.74**			**0.52**				**0.09**			**0.25**
6 – 9	-	-	-		-	-		5.23	Ref			-	-	
10 – 13	-	-	-		-	-		5.24	0.01	-0.58 – 0.59	0.99	-	-	
14 – 17	8.74	Ref			Ref			6.00	0.77	0.13 – 1.41	0.02	Ref		
18 – 25	8.97	0.23	-0.59 – 1.04	0.59	0.36	-0.47 – 1.19	0.40	5.33	0.10	-0.52 – 0.72	0.76	-0.56	-1.25 – 0.12	0.10
>25	8.75	0.01	-0.76 – 0.77	0.99	0.53	-0.42 – 1.48	0.27	5.24	0.01	-0.55 – 0.56	0.99	-0.43	-1.21 – 0.36	0.29

**Gender**														
Female	8.83	Ref			Ref			5.28	Ref			Ref		
Male	8.79	-0.04	-0.60 – 0.52	0.88	-0.13	-0.69 – 0.43	0.64	5.49	0.21	-0.18 – 0.59	**0. 29**	-0.28	-0.74 – 0.19	**0.24**
**History of schistosomiasis**														
No	8.50	Ref			Ref			4.92	Ref			Ref		
Yes	9.32	0.82	0.29 – 1.35	**0.003**	0.90	0.35 – 1.45	0.001	6.15	1.23	0.85 – 1.60	**<0.001**	0.77	0.31 – 1.22	**<0.001**
**Location**				**0.004**			**0.005**				**<0.001**			**<0.001**
Lambaréné	9.22	Ref			Ref			4.78	Ref			Ref		
Zilé-PK	8.07	-1.14	-1.78 – -0.51	<0.001	-1.34	-2.11 – -0.57	<0.001	6.41	1.62	1.21 – 2.03	<0.001	0.77	0.14 – 1.41	0.02

Mitonè-PK	8.58	-0.64	-1.59 – 0.32	0.19	-0.80	-1.89 – 0.30	0.15	7.29	2.50	1.88 – 3.13	<0.001	2.01	1.11 – 2.92	<0.001
Bindo-Makouké	8.49	-0.73	-1.57 – 0.11	0.09	-0.81	-1.75 – 0.14	0.09	4.37	-0.42	-0.98 – 0.15	0.15	-1.36	-2.14 – -0.57	<0.001
**Educational level**				**0.004**			**0.06**				**0.15**			**0.005**
Primary	8.09	Ref			Ref			5.49	Ref			Ref		
Secondary	9.10	1.01	0.41 – 1.61	0.001	0.79	0.11 – 1.47	0.02	5.28	-0.21	-0.59 – 0.17	0.28	-0.41	-0.97 – 0.15	0.15
Other	8.64	0.55	-0.77 – 1.88	0.41	0.52	-0.81 – 1.85	0.44	4.57	-0.92	-1.93 – 0.08	0.07	-1.76	-2.86 – -0.66	0.002
**Family size**				**0.11**			**0.02**				**0.20**			**0.007**
1 – 3	8.36	Ref			Ref			5.61	Ref			Ref		
4 – 6	9.07	0.71	0.04 – 1.37	0.04	0.86	0.20 – 1.52	0.01	5.14	-0.47	-1.08 – 0.13	0.12	-0.70	-1.24 – -0.15	0.01

7 – 25	8.85	0.48	-0.21 – 1.18	0.17	0.75	0.04 – 1.47	0.04	5.55	-0.06	-0.69 – 0.57	0.84	-0.87	-1.47 – -0.28	0.004
**Use private toilets at home**														
No	8.74	Ref			Ref			5.54	Ref			Ref		
Yes	9.38	0.64	-0.15 – 1.43	**0.11**	0.27	-0.60 – 1.14	0.55	4. 06	-1.48	-2.05 – -0.90	**<0.001**	-0.28	-1.01 – 0.43	**0.42**
**Use pit latrine at home**														
No	9.11	Ref			Ref			4.79	Ref			Ref		
Yes	8.59	-0.51	-1.04 – 0.01	**0.06**	-0.21	-0.82 – 0.40	0.50	5.58	0.79	0.38 – 1.20	**<0.001**	-0.12	-0.62 – 0.38	**0.63**
**Home proximity with water course**														
No	8.85	Ref			Ref			4.96	Ref			Ref		

Yes	8.81	-0.03	-0.60 – 0.53	**0.90**	-0.12	-0.73 – 0.49	0.70	5.55	0.59	0.19 – 0.99	**0.004**	0.03	-0.47 – 0.53	**0.89**
**Use fresh water**														
No	9.13	Ref			Ref			4.43	Ref			Ref		
Yes	8.68	-0.45	-1.02 – 0.11	0.11	0.14	-0.56 – 0.84	0.70	5.79	1.36	0.97 – 1.75	**<0.001**	0.79	0.21 – 1.37	**0.008**
**Main occupation**				**0.05**			**0.12**				**0.36**			**0.93**
None	8.45	Ref			Ref			5.16	Ref			Ref		
Student	9.11	0.65	-0.08 – 1.38	0.08	0.69	-0.13 – 1.52	0.10	5.61	0.45	-0.22 – 1.12	0.19	0.20	-0.48 – 0.88	0.56
Farmer/Fisher	8.39	-0.06	-0.83 – 0.70	0.78	0.67	-0.18 – 1.52	0.12	5.68	0.52	-0.18 – 1.22	0.14	-0.12	-0.82 – 0.58	0.73

Trader	8.62	0.17	-0.97 – 1.30	0.77	0.01	-1.12 – 1.14	0.98	5.38	0.22	-0.82 – 1.26	0.68	-0.14	-1.08 – 0.79	0.76
Other	9.41	0.95	0.17 – 1.73	0.02	0.87	0.08 – 1.66	0.03	5.08	-0.08	-0.80 – 0.63	0.82	-0.06	-0.72 – 0.58	0.84

*Assessed among youg adults and adults who responded to the main study questionnaire; α: Coefficient of regression estimating the difference in mark between the associated modality and the modality of reference of the variable; $^{\beta}$The model of the adjusted analysis included all data considered for crude analysis
